# Diabetes Is Associated with Worse Clinical Presentation in Tuberculosis Patients from Brazil: A Retrospective Cohort Study

**DOI:** 10.1371/journal.pone.0146876

**Published:** 2016-01-11

**Authors:** Leonardo Gil-Santana, Jilson L. Almeida-Junior, Carolina A. M. Oliveira, Lucas S. Hickson, Carla Daltro, Simone Castro, Hardy Kornfeld, Eduardo M. Netto, Bruno B. Andrade

**Affiliations:** 1 Unidade de Medicina Investigativa, Laboratório Integrado de Microbiologia e Imunorregulação, Centro de Pesquisas Gonçalo Moniz, Fundação Oswaldo Cruz, Salvador, Bahia, Brazil; 2 School of Medicine, Faculdades de Tecnologia e Ciências, Salvador, Bahia, Brazil; 3 Multinational Organization Network Sponsoring Translational and Epidemiological Research (MONSTER) Initiative, Fundação José Silveira, Salvador, Brazil; 4 Research Center, Instituto Brasileiro para Investigação da Tuberculose (IBIT), Fundação José Silveira, Salvador, Bahia, Brazil; 5 Hospital Universitário Professor Edgard Santos, Universidade Federal da Bahia, Salvador, Bahia, Brazil; 6 Department of Medicine, University of Massachusetts Medical School, Worcester, Massachussetts, United States of America; University of Cape Town, SOUTH AFRICA

## Abstract

**Background:**

The rising prevalence of diabetes mellitus (DM) worldwide, especially in developing countries, and the persistence of tuberculosis (TB) as a major public health issue in these same regions, emphasize the importance of investigating this association. Here, we compared the clinical profile and disease outcomes of TB patients with or without coincident DM in a TB reference center in Brazil.

**Methods:**

We performed a retrospective analysis of a TB patient cohort (treatment naïve) of 408 individuals recruited at a TB primary care center in Brazil between 2004 and 2010. Data on diagnosis of TB and DM were used to define the groups. The study groups were compared with regard to TB disease presentation at diagnosis as well as to clinical outcomes such as cure and mortality rates upon anti-tuberculosis therapy (ATT) initiation. A composite score utilizing clinical, radiological and microbiological parameters was used to compare TB severity between the groups.

**Results:**

DM patients were older than non-diabetic TB patients. In addition, diabetic individuals more frequently presented with cough, night sweats, hemoptysis and malaise than those without DM. The overall pattern of lung lesions assessed by chest radiographic examination was similar between the groups. Compared to non-diabetic patients, those with TB-diabetes exhibited positive acid-fast bacilli in sputum samples more frequently at diagnosis and at 30 days after ATT initiation. Notably, higher values of the TB severity score were significantly associated with TB-diabetes comorbidity after adjustment for confounding factors. Moreover, during ATT, diabetic patients required more frequent transfers to TB reference hospitals for complex clinical management. Nevertheless, overall mortality and cure rates were indistinguishable between the study groups.

**Conclusions:**

These findings reinforce the idea that diabetes negatively impacts pulmonary TB severity. Our study argues for the systematic screening for DM in TB reference centers in endemic areas.

## Introduction

The association between diabetes mellitus (DM) and tuberculosis (TB) has been known since the beginning of the 20th century [1]. However, it was only after the recent increase in the burden of type 2 diabetes [2], attributed mainly to the modern lifestyle changes, that the link between these two diseases was subject to further interest. According to the World Health Organization (WHO), an estimated 350 million people around the world had diabetes in 2011, 80% of whom were living in developing countries. Notably, it is predicted that global diabetes prevalence will increase 50% by 2030 with the greatest increase in low and middle income countries [3]. Despite the decrease of mortality observed in the last two decades, Brazil remains among the 22 countries responsible for 80% of TB cases worldwide with a death rate of 2.3 per 100 thousand inhabitants, [4]. Scarce data have been reported on the prevalence of DM in Brazil. The prevalence of DM was estimated to be 7.6% in a cross-sectional home survey conducted from November 1986 to July 1988 in a random sample of 21,847 individuals aged 30–69 years in nine Brazilian cities [5]. Importantly, a recent study demonstrated that the proportion of diabetes among TB cases in Brazil increased from 380 cases/100,000 habitants to 6,150/100,000 between 2001 to 2011 [6], highlighting the need for more systematic studies describing epidemiology and clinical aspects of TBDM comorbidity in the country.

Diabetes is linked to increased risk for certain infectious diseases including TB [1,7]. We have recently shown that patients with TBDM comorbidity from South India display heightened levels of plasma biomarkers of inflammation, tissue remodeling, and oxidative stress; all of which could be driving increased susceptibility to worse TB-related clinical outcomes [8]. Understanding TBDM interaction in Brazil could provide a basis in evidence for enhanced clinical management of patients with this dual burden and have a positive impact on public health.

The present study compares the clinical presentation profile and outcomes of TB in diabetic and non-diabetic patients recruited at a TB primary care reference center in a highly TB endemic area in Brazil [9]. The results indicate that diabetes may exacerbate TB disease severity.

## Methods

### Ethics statement

All clinical investigations were conducted according to the principles expressed in the Declaration of Helsinki. The study was approved by the Ethics Committee of the Maternidade Clímério de Oliveira, Federal University of Bahia (protocol number: 037/2011, Ethics committee approval number: 034/11). All materials given to the research team were de-identified.

### Study site

The study was performed at the Instituto Brasileiro para a Investigação da Tuberculose (IBIT, Brazilian Institute for TB investigation), which is a philanthropic TB outpatient clinic in Salvador, Brazil. This institute is part of the Brazilian National TB control program and has been providing free TB diagnosis, clinical care, anti-microbial chemotherapy and social support services to TB patients since its foundation on 1937. IBIT serves as a reference center for TB primary care and annually treats 10–15% TB cases of the city of Salvador, Bahia [10]. Diagnosis of TB at IBIT follows the guidelines of the Brazilian Society of Pulmonology and Tisiology [11], which is similar to WHO recommendations [12]. ATT is also performed following the Brazilian national guidelines [11]. All patients who receive a TB diagnosis at IBIT are included sequentially in a registry and followed monthly until cure or transfer to tertiary care centers (TB reference hospitals), which occurs in the case of medical complications that require more complex clinical management. In the circumstance of a patient being transferred to tertiary centers, follow-up information, including those directly related to death, treatment failure and/or relapse, were electronically available for the study through a county surveillance system. During each patient visit, specialized physicians complete clinical report forms with information on socio-economic status, epidemiological and clinical features. Comorbidities such as HIV infection, cancer, hypertension and metabolic diseases are actively screened at routine patient visits. Radiological evaluation is also systematically performed in all patients. IBIT’s nurses trained in public health and data management input all the information collected during the patient visits into IBIT’s electronic databank, which is used to assist the health personnel involved with patient care. At the time of this study, all identifying patient information was either coded or redacted by IBIT.

### Study design

We performed a retrospective analysis of medical records including all TB cases seen at IBIT from 2004 to 2010, a total of 2,189 individuals. In the patient datasets, we searched for those who, at the time of TB diagnosis (treatment naïve), had DM diagnosis by either self-report or registry of DM diagnosis criteria following the American Diabetes Association guidelines [13] in the medical reports. We identified 135 individuals with TB-diabetes. Moreover, we sampled TB patients who did not have diagnosis for DM in the clinical report forms. We systematically assigned individuals enrolled in IBIT’s registry book immediately prior to or after a TBDM individual was enrolled. These individuals are identified in this study as non-DM TB patients. In the case that a non-DM TB individual was non-eligible a person listed immediately prior or later was selected instead. Following these criteria, we selected 273 non-DM TB individuals. Exclusion criteria for this study were age under 18-years old and incomplete medical reports. In order to minimize information bias, the data used in this particular study were collected utilizing standardized questionnaires, which were filled by two of the authors (C.A.M.O. and L.S.H.).

### TB severity score

In order to estimate TB disease severity in our study population, we used a modified version of a previously published clinical score [14]. In this scoring system, one point was given for the presence of each major TB-related symptom (cough, fever, weigh loss, night sweats, anorexia, hemoptysis and malaise). The total score can range from zero (no symptoms) to 7 (all symptoms) [14]. In this context, patients presenting with a score value ≥4 were classified as having severe TB. In addition, we built a composite score combining the same TB symptoms (cough, fever, weigh loss, night sweats, anorexia, hemoptysis and malaise), one radiographic finding in posteroanterior chest x-ray (presence of cavitary lesion) assessed by an expert physician in the clinic (E.M.N.) and presence of acid-fast bacilli (AFB) positive sputum smear at TB diagnosis. To produce the score value, one point was given for the presence of each symptom, cavitary TB, and positive AFB smear. Thus, the total score could range from zero (no symptoms detected, no cavitary lesion and AFB negative sputum smear at diagnosis) to 9 (all symptoms, presence of cavity by chest radiography and AFB positive smear). Individuals were further categorized as having mild TB (scores from zero to 5) or severe TB (score >5).

### Data analysis

The median values with interquartile ranges (IQR) were used as measures of central tendency. The Fisher’s and chi-square tests were used to compare nominal variables between diabetic and non-diabetic individuals with TB. Continuous variables were compared between the study groups using the Mann-Whitney test. Logistic regression analyses adjusted for age and gender were performed to assess the odds ratios (OR) of the associations between the TB severity score and TB-diabetes comorbidity. Multinomial logistic regression analysis adjusted for age and gender was used to assess the odds ratios of the associations between TB-diabetes comorbidity and transfer to a tertiary health care center. A p-value below 0.05 was considered statistically significant. The statistical analyses were performed using SPSS 20.0 (IBM statistics), Graphpad Prism 6.0 (GraphPad Software, San Diego, CA) and JMP 10.0 (SAS, Cary, NC, USA).

## Results

By retrospectively assessing clinical report forms from our TB cohort, we detected 135 diabetic patients (TBDM) and selected 273 non-DM individuals. Gender distribution was similar between the groups (male gender = 64.4% in TB vs. 54.8% in TBDM; P = 0.11). TBDM individuals were on average older than non-diabetics (median age: 37.9 yrs., IQR: 27.3–49.6 vs. 52 yrs., IQR: 45.9–60, P<0.001; Table 1). In addition, both groups displayed comparable BMI values (median BMI = 19.68 kg/m^2^; IQR: 17.16–21.40 vs. 21.36 kg/m^2^; IQR: 20.04–24.95; P = 0.43). Moreover, TBDM and non-DM TB patients did not differ with regard to lifestyle habits (chronic alcoholism, smoking and use of illicit drugs), prevalence of HIV and/or cancer comorbidities, and prevalence of tuberculin skin test (TST) positivity (Table 1).

**Table 1:** Characteristics of the study participants.

Characteristic	(TB / TBDM)	TB	TBDM	P-value
**Male–n (%)**	273 / 135	173 (63.4)	74 (54.8)	0.11
**Median age–y (IQR)**	273 / 135	37.9 (27.3–49.6)	52 (45.9–60)	<0.001
**Median BMI–kg/m**^**2**^ **(IQR)**	68 / 40	19.7 (17.2–21.4)	21.4 (20–24.9)	0.43
**Chronic alcoholism–n(%)**	263 / 128	53 (20.2)	19 (14.8)	0.21
**Smoking–n(%)**	265 / 134	114 (43.0)	65 (48.5)	0.33
**Illicit drugs use–n(%)**	267 / 132	6 (5.9)	3 (2.2)	0.13
**HIV/AIDS–n(%)**	269 / 132	2 (0.7)	0 (0)	0.32
**Cancer–n(%)**	269 / 132	4 (1.5)	1 (0.8)	0.54
**TST result–n (%)**	189 / 85			0.96
< 5 mm		10 (5.3)	5 (5.9)	
5 to 10 mm		10 (5.3)	4 (4.7)	
≥ 10 mm		169 (89.4)	76 (89.4)	

Frequency data were compared using the exact Fisher’s test and Chi-square (for TST result) whereas continuous variables (age and BMI) were compared using the Mann-Whitney test. The TB / TBDM column shows number of patients in each study group that information was available for. BMI, Body Mass Index; DM, Diabetes Mellitus; IQR, Interquartile range; TB, Tuberculosis; TST, tuberculin skin test.

**Fig 1:**
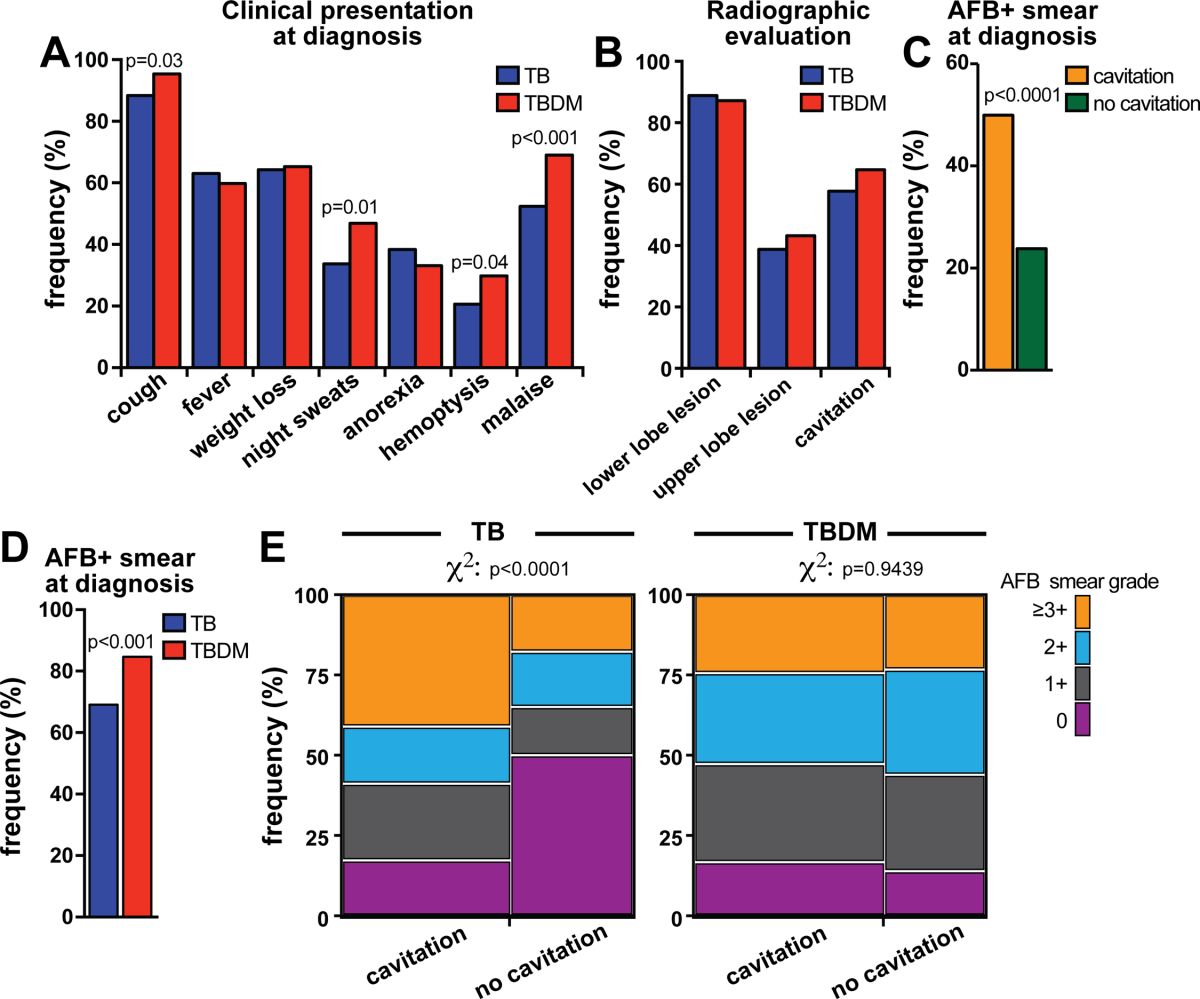
Clinical, radiographic and microbiological profiles of patients with TB-diabetes comorbidity. (A) Prevalence of indicated TB-related symptoms were compared between patients with TB-diabetes (TBDM) comorbidity and non-diabetics (TB). (B) Frequency of lung lesions identified by chest radiography was compared between the study groups. Prevalence of AFB positive smear in TB patients with or without lung cavitary lesions (C) and in diabetic or non-diabetic patients (D) is shown. (E) AFB distribution profile in patients with or without cavitary lung lesion from either TB without DM or in TBDM patients. The Fisher’s exact test was used to assess statistical significance in in (A-D) whereas the chi-square test was used to compare data in (E).

We next compared the groups with regard to clinical presentation of TB disease. Interestingly, only one patient with DM (0.7%) presented with extra-pulmonary TB (pericardial TB), compared to 19 (7%) in non-DM TB patients (Table 1). Among non-DM TB patients, the extra-pulmonary sites were pleura (n = 19, 67.8%), lymph nodes (n = 7, 25.0%), larynges (n = 1, 3.6%) and eyes (n = 1, 3.6%). Symptoms that are classically associated with TB (cough, night sweats, hemoptysis, and malaise) [4,14] were more frequent in TBDM patients (Fig 1A and Table 2). Radiological evaluation revealed that non-DM TB patients exhibited similar distribution of lung lesions and prevalence of cavitation than those with TBDM comorbidity (Fig 1B). In the entire study population, cavitary TB was associated with higher frequency of individuals presenting with positive AFB smear samples (49.9% of AFB+ in cavitary TB vs. 23.8% in non-cavitary disease, p<0.0001; Fig 1C).

**Table 2:** Clinical and laboratory characterization of the study participants.

Variables–n (%)	(TB / TBDM)	TB	TBDM	P-value
**Positive sputum smear**				
Pre-ATT	269 / 131	186 (69.1)	111 (84.7)	<0.0001
30 days of ATT	75 / 44	11 (14.7)	18 (40.9)	0.01
60 days of ATT	118 / 74	13 (10.9)	6 (8.1)	0.71
180 days of ATT	214 / 105	8 (3.8)	2 (2)	0.68
**Positive culture pre-ATT**	193 / 81	164 (85)	73 (90.1)	0.27
**Extrapulmonary TB**	273 / 135	19 (7)	1 (0.7)	<0.0001

Frequency data were compared using the exact Fisher’s test. DM, Diabetes Mellitus; TB, Tuberculosis. The TB / TBDM column shows number of patients in each study group that information was available for.

Smear positive TB cases are thought to transmit *M*. *tuberculosis* more efficiently than those with smear negative screening [15]. In addition, previous studies have indicated that smear positive TB patients exhibit higher levels of acute phase protein in blood as well as bilateral lung lesions [16], indicating that presence of *M*. *tuberculosis* in sputum may be associated with more severe forms of TB disease. In our study, the proportion of patients with an initial positive AFB sputum smear was greater amongst the TBDM group (84.7% vs. 69.1%, p<0.001; Fig 1D). Notably, after 30 days of ATT initiation, positive smears were more frequent in TBDM than non-DM TB individuals (40.9% vs. 14.7%; p = 0.01; Table 2). We next asked whether presence of cavitary lung lesions was associated with increased bacterial loads of sputum samples in both TBDM and non-DM pulmonary TB patients. Non-diabetic individuals presenting with cavitary TB exhibited increased frequency of positive AFB smear detection and also higher bacterial loads (e.g. AFB ≥3+) compared to non-diabetic individuals without lung cavities (chi-square P<0.0001; Fig 1E). Intriguingly, we observed that in TBDM patients, cavitary TB was not associated with further increases in bacterial loads assessed by sputum AFB stain (chi-square p = 0.9439; Fig 1E). These observations suggest that at the time of TB diagnosis, DM patients might have greater lung bacterial loads than non-DM or that DM causes a unique, non-cavitary pathology that enbales *M*. *tuberculosis* to reach the airways.

**Fig 2:**
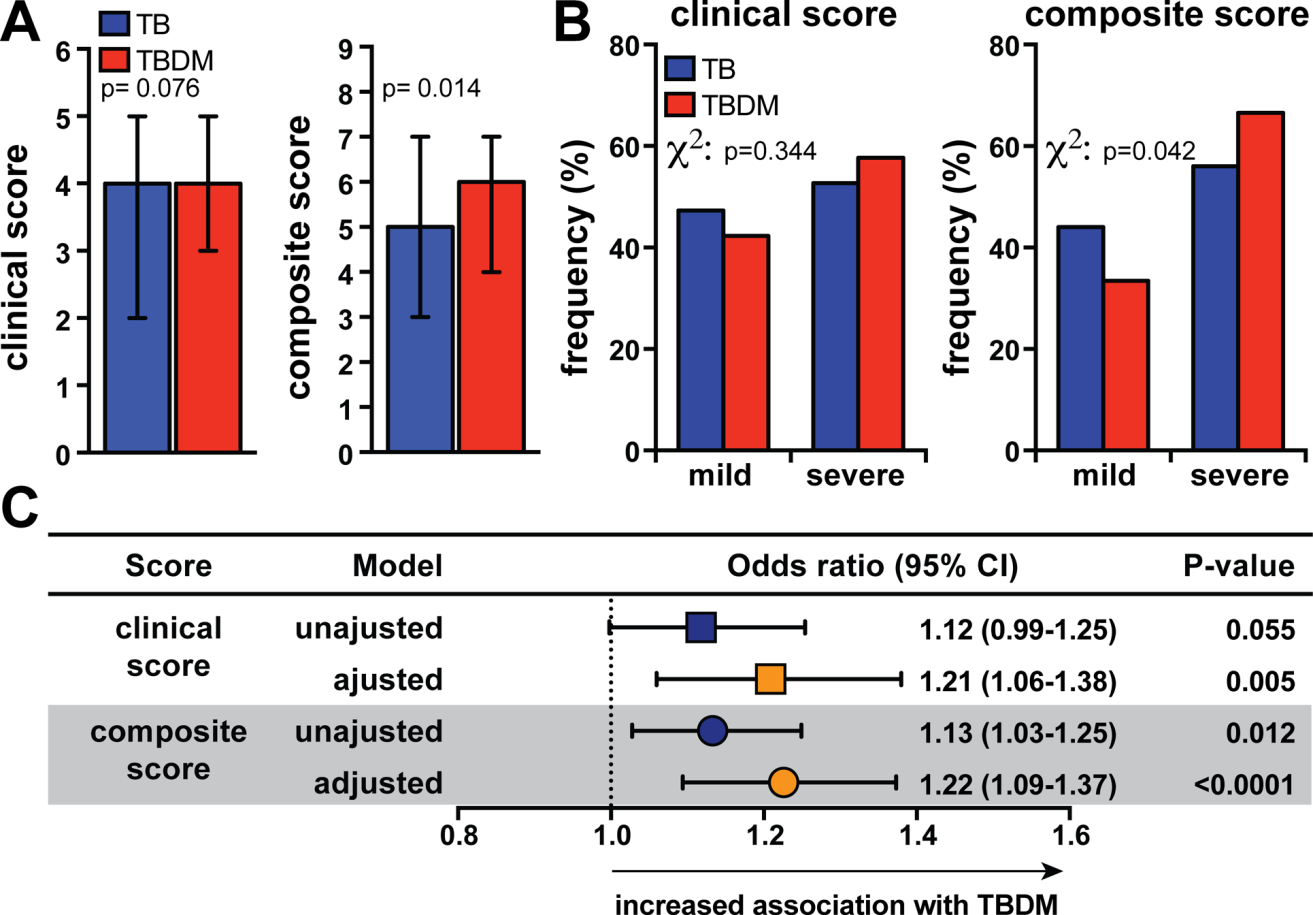
TB-diabetes comorbidity is associated with increased TB severity assessed by a composite score. (A) The differences in median values (and IQR) between clinical score (left panel; only symptoms were considered) and composite score (right panel; symptoms, presence of cavitation and AFB+ in smear samples were considered) obtained in TB and TBDM groups were compared using the Mann-Whitney test. (B) Patients were further categorized in mild TB (<4points) or severe TB (≥4 points) using the clinical score (left panel). A similar stratification in mild TB (≤5 points) or severe TB (>5 points) was performed using the composite score. The frequency profile of TB and TBDM patients classified into severity categories was compared using the Fisher’s exact test. (C) Linear regression analysis were compared for clinical and composite score adjusted for age and gender was utilized to determine the association between increases of 1 point in the severity scores and presence of TB-diabetes comorbidity (OR, Odds ratio; 95%CI, 95% confidence interval).

Clinical scores designed to infer TB disease severity have been previously reported [14,17,18]. Here, we composed a score adapted from previous work [14], utilizing clinical symptoms shown in Fig 1A. In addition, we created a new score considering also radiological (presence of cavitation) and microbiological (AFB smear positivity) information at time of diagnosis. When the purely clinical score is applied, we failed to observe differences in the values between TB and TBDM patients (Fig 2A). On the other hand, analysis using the composite score with radiological and microbiological data revealed that TBDM individuals displayed higher values than non-DM TB patients (Fig 2A). We further categorized individuals into groups of mild and severe TB based on the prevalence of symptoms, lung cavitation and smear positivity. Once again, stratification of patients using of the clinical score alone could not discriminate mild (<4 points) from severe TB (≥4 points) between TB and TBDM (Fig 2B). Using the expanded composite score, TBDM comorbidity was associated with increased frequency of patients presenting with severe disease (Fig 2B). Importantly, higher values of the composite score were associated with occurrence of TBDM even after adjustments for potential confounding factors, such as age and gender (Fig 2C). These findings argue that DM affects TB clinical presentation, possibly driving more severe forms of this disease. In addition, we conclude that our composite score may be better discriminator of TB disease severity in TBDM than purely clinical scores.

**Fig 3:**
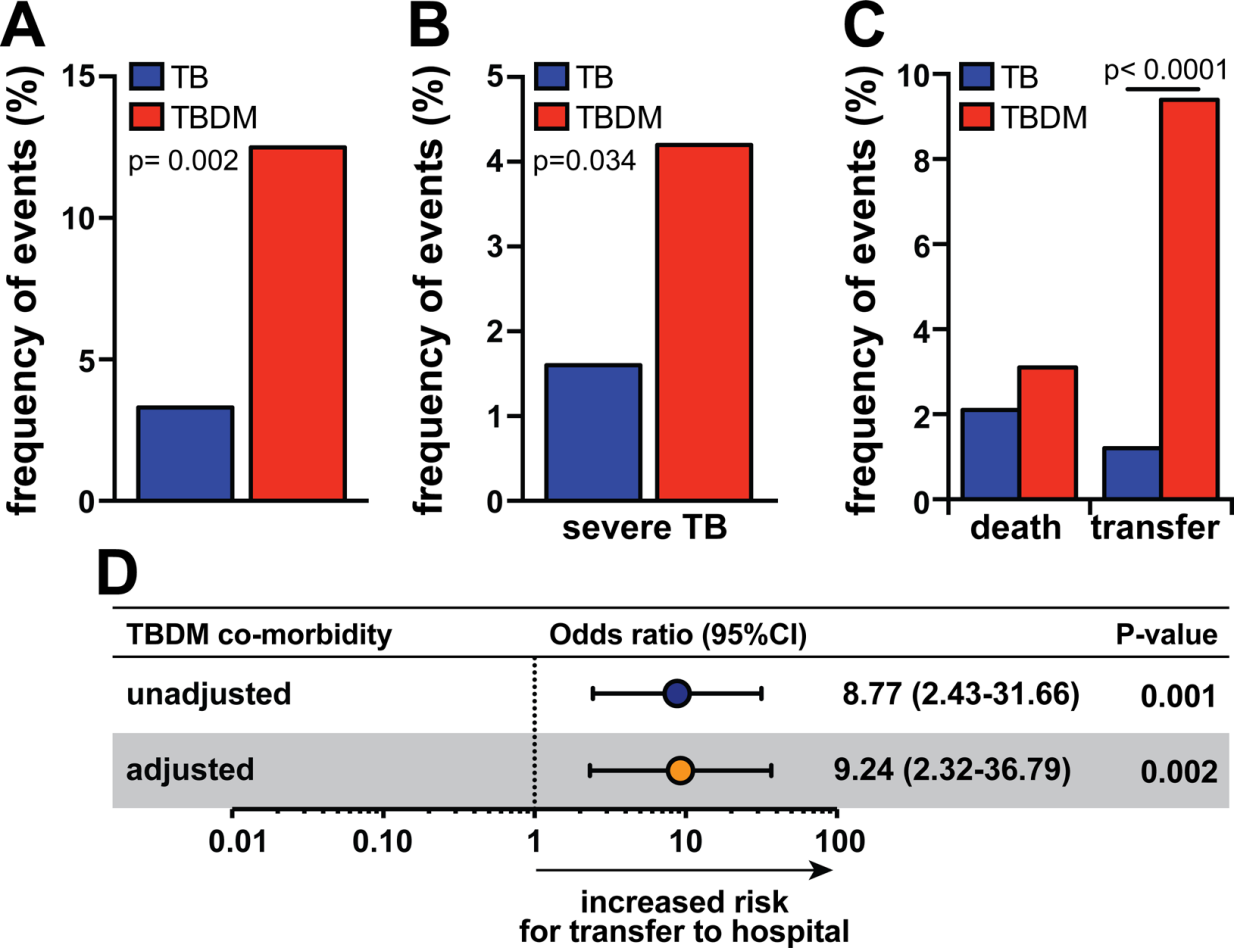
TB-diabetes comorbidity is associated with higher frequency of patient transfer to tertiary health care centers. (A) Frequencies of total undesirable events upon ATT initiation (death and transference to tertiary health care centers) in patients with TB or TBDM. (B) Frequencies of total undesirable events upon ATT initiation between patients with TB or TBDM with severe TB disease according to a composite score. (C) Frequency of death or transfer to tertiary health care centers in TB and TBDM patients. (D) Multinomial logistic regression analysis adjusted for age and gender was used to test association between TBDM comorbidity and the transfer to tertiary health care centers (OR, Odds ratio; 95%CI, 95% confidence interval). In (A) and (C), data were compared using the Fisher’s exact test. In (B), data were compared using the chi-square test.

Both patient groups displayed similar cure rates with antimicrobial therapy (TB: 93.7% of cure vs. TBDM: 85.5%, p = 0.72; Table 3). Nevertheless, TBDM patients experienced unfavorable events more frequently after treatment initiation (Fig 3A), which were defined here as death or transfer to TB reference hospitals for more complex health care. Frequency of these events was higher TBDM patients presenting with severe disease (Fig 3B). TBDM patients were transferred more frequently to TB reference hospitals (Fig 3C) but exhibited similar mortality rates to non-DM TB individuals (Fig 3C). Of note, all patients with extrapulmonary TB and the two HIV-infected individuals included in the present study did not exhibit unfavorable events (all patients cured from TB after treatment and did not required transfer to a tertiary center). Multinomial logistic regression analysis adjusted for age and gender confirmed the association between occurrence of TBDM comorbidity and the need of transfer to tertiary care centers (Fig 3D). These results reinforce the idea that coincident TBDM is associated with more severe TB presentation.

**Table 3:** Clinical outcomes of the study participants upon anti-tuberculosis treatment initiation.

Outcome	TB (n = 244)	TBDM (n = 128)	P-value
**Cure**	236 (93.7)	112 (85.5)	0.72
**Death**	5 (2)	4 (3.1)	0.44
Transfer*	3 (1.1)	12 (9.2)	<0.0001

*Transfer occurred due to uncontrollable disease in the clinic. DM, Diabetes Mellitus; TB, Tuberculosis.

## Discussion

Understanding the impact of DM on TB susceptibility in countries that suffer from the dual burden of these diseases will be key to ushering in focused control strategies adapted to local epidemiological trends. The present study describes clinical, radiographic and microbiological features of individuals with TBDM comorbidity compared to non-DM TB patients recruited at a tuberculosis primary care reference center in Brazil, which displays one of the highest treatment-driven TB cure rates in the country [10].

The prevalence of non-communicable diseases, including DM, increases with age [19,20]. Our finding that TBDM patients were on average older than non-diabetic TB individuals reflects at pattern. According to WHO data, there is a higher prevalence of TB in young adults, especially males [21]. The association between gender and DM seems to be more complex as differences in lifestyle may cause changes in the risk of developing DM, influencing the prevalence of this disease in women and men. We found that a majority of the pulmonary TB population evaluated herein were men; however we could not detect differences in gender distribution between TBDM and non-DM TB cases. More importantly the combined effects of age, gender, and additional risk factors for DM, such as obesity, may lead to differential susceptibility for development of symptomatic DM. In the present study the non-DM TB population had BMI values within the normal range (>18.5 Kg/m^2^) and although TBDM individuals exhibited slightly higher values than non-DM, this difference was not statistically significant. Our outpatient clinic center is designed to provide primary TB care and thus the patient population may not have presented with advanced TB-driven consumption syndrome, which would possibly explain why most of the individuals had BMI within the normal limits.

There are several indications that clinical presentation of TB disease is exacerbated in individuals with poor glycemic control [22,23]. In addition, glycemic control is thought to influence radiographic manifestation of pulmonary TB in patients with DM [23]. In agreement with these studies, we found that compared to non-DM TB individuals TBDM patients more frequently exhibited symptoms classically associated with pulmonary *M*. *tuberculosis* infection including cough, night sweats, hemoptysis and malaise. DM has been associated with increased frequency of atypical radiological findings as well and cavitary TB [24–26]. Intriguingly, the study groups assessed here did not differ in terms of radiographic localization of lung lesions or the presence of cavitation, suggesting that the impact of DM on TB-associated lung tissue destruction in our patient population was subtle. The reasons behind this discrepancy may involve differences in the populations studied and/or primary care vs. hospital-based settings. Nevertheless, we observed that TBDM was associated with higher frequency of AFB positive sputum smear samples at the time of diagnosis and remained increased up to 30 days after ATT initiation. However, these differences were no longer observed at later time points (day 60 and 180) after treatment initiation. If our results showing that TBDM is associated with more AFB+ at diagnosis and delayed sputum conversion are validated in future studies, then DM might be disproportionately contributing to transmission within a community, making this condition a problem for TB control across the whole population and not only the TB cases.

We also noticed that presence of cavitation was associated with AFB positivity in sputum smears regardless of DM status. We performed a further sub-analysis stratifying the clinical groups per AFB sputum grade and presence of cavitation in chest X-rays. As expected in non-DM TB individuals, the frequency of higher AFB sputum grades was increased in individuals with lung cavitation compared to those without pulmonary lesions. Strikingly, in the DM subpopulation, presence of cavities was no longer associated with higher AFB smear grades, indicating that this type of lung lesion is not a predisposing factor driving higher *M*. *tuberculosis* loads observed in diabetes. Moreover, our findings indicate that the pathophysiology of TB in the context of DM may be restricted to the lungs, as we observed a significantly lower prevalence of extrapulmonary TB cases in TBDM patients compared to non-diabetics. Decreased frequency of extrapulmonary TB in diabetic patients has been previously reported in studies from India [27,28] and our study reinforces this idea also in a Brazilian cohort. This phenomenon is the opposite of what occurs in HIV-TB co-infected patients and patients treated with TNF inhibitors, where extrapulmonary TB is disproportionally increased [29,30]. The mechanisms underlying the discrepancies reported here involving an apparent restriction of susceptibility to pulmonary TB, increased *M*. *tuberculosis* loads and cavitary lesion formation in TB-DM comorbidity deserve further investigation.

Clinical scores are useful tools to systematically assess TB severity. Here, we adapted a previously published clinical score [14] and extended its composition to include radiographic (presence of lung cavitation on the chest X-ray) and microbiological (presence of AFB positive sputum smear) characteristics at the diagnosis (pre-ATT). We speculate that the severity score proposed here increases sensitivity to detect differences caused by the impact of DM on TB because it incorporates variables linked to TB transmissibility (cavitary lesion and AFB positive smear), which should be considered in public health policy. We found that TBDM individuals display higher TB severity assessed by this composite score (which was not detected when only a clinical score was used), further highlighting that the detrimental association between DM and TB.

Several studies have demonstrated that coincident DM increases the risk of TB treatment failure, death, and an overall higher frequency of adverse events [23,31–33], including in Brazil [6]. Our findings showing that clinical management of TBDM individuals required a greater number of patient transfers to tertiary care centers, implies worse clinical prognosis in TBDM. This scenario, together with the lack of efforts from the Brazilian health authorities to provide special care for management of TBDM cases, could amplify the cost of public health care. A recent systematic review has pointed out the urgent need of increased attention to treatment of TB in people with DM [23]. This may include routine testing of suspected DM, improved glycaemia control and increased clinical and therapeutic monitoring.

Although our results showing increased TB severity in diabetic individuals are in agreement with several other studies [7,32–34], this finding has not been universal. Some studies have shown that clinical characteristics of TB do not significantly differ between DM and non-DM patients (reviewed in [35]). Additional systematic analyses of studies from different countries and diverse epidemiological settings are necessary to better delineate the interaction between TB and DM worldwide.

The results presented here are subject to common limitations of retrospective studies with regard to the documentation of diagnosis and conditions. Particularly concerning the diagnosis of DM, our criteria were self-disclosure of the condition or laboratory results consistent with the DM diagnosis following the American Diabetes Association criteria [18]. It is possible that some diabetic patients were not identified at the time of TB diagnosis because of an incomplete investigation. Furthermore, we used AFB grade to infer bacterial loads but this parameter is less accurate than month 2 conversion or time to culture positivity. We did not have systematic documentation of culture conversion in the clinical reports assessed in the present study. We also did not have data concerning the status of glycemic control in diabetic patients, which could affect the impact of DM in TB presentation. We did not find differences in the classical outcomes systematically examined by some other studies such as death, relapse or reinfection, possibly due to characteristics of the patients recruited at our primary care reference center. Finally, in our cohort, the number of unfavorable events was small, which may reflect the high cure rates (≥90%) documented in our institution [10]. A prospective validation study to further evaluate the association of these two diseases in the current epidemiological setting is therefore warranted.

## Conclusions

The present study performed in a primary TB care reference center in Brazil, reinforces the argument that DM negatively impacts the TB clinical profile. Active DM detection in TB reference centers in endemic countries requires systematic implementation to identify population at higher risk of adverse events after ATT initiation (such as transfer to tertiary centers) and potentially reduce the cost of patient care and TB transmission. Our findings also raise the necessity of a greater investigation and control of DM in the general population of TB endemic countries.

## References

[1] Dooley KE, Chaisson RE (2009) Tuberculosis and diabetes mellitus: convergence of two epidemics. Lancet Infect Dis 9: 737–746. 10.1016/S1473-3099(09)70282-8 19926034 PMC2945809

[2] Wild S, Roglic G, Green A, Sicree R, King H (2004) Global prevalence of diabetes: estimates for the year 2000 and projections for 2030. Diabetes Care 27: 1047–1053. 15111519 10.2337/diacare.27.5.1047

[3] International Diabetes Federation (2013) IDF Diabetes Atltas 6^th^ edition. www.ifd.org/diabetesatlas (assessed on 10/15/2015).

[4] World Health Organization (2014) Global tuberculosis report. http://www.who.int/tb/publications/global_report/en/ (assessed on 07/09/2015).

[5] Malerbi DA, Franco LJ (1992) Multicenter study of the prevalence of diabetes mellitus and impaired glucose tolerance in the urban Brazilian population aged 30–69 yr. The Brazilian Cooperative Group on the Study of Diabetes Prevalence. Diabetes Care 15: 1509–1516. 1468278 10.2337/diacare.15.11.1509

[6] Reis-Santos B, Gomes T, Locatelli R, de Oliveira ER, Sanchez MN, Horta BL, et al (2014) Treatment outcomes in tuberculosis patients with diabetes: a polytomous analysis using Brazilian surveillance system. PLoS One 9: e100082 10.1371/journal.pone.0100082 25003346 PMC4086729

[7] Jeon CY, Murray MB (2008) Diabetes mellitus increases the risk of active tuberculosis: a systematic review of 13 observational studies. PLoS Med 5: e152 10.1371/journal.pmed.0050152 18630984 PMC2459204

[8] Andrade BB, Pavan Kumar N, Sridhar R, Banurekha VV, Jawahar MS, Nutman TB, et al (2014) Heightened plasma levels of heme oxygenase-1 and tissue inhibitor of metalloproteinase-4 as well as elevated peripheral neutrophil counts are associated with TB-diabetes comorbidity. Chest 145: 1244–1254. 10.1378/chest.13-1799 24458266 PMC4042512

[9] Ministério da Saúde, Brasil (2002) Programa Nacional de Controle da Tuberculose. http://bvsms.saude.gov.br/bvs/publicacoes/ProgramaTB.pdf (assessed on 10/07/2015).

[10] Slatery E (2014) Neglected or Non-compliant? Assessing the difficulties of tuberculosis patients in Salvador-BA, Brazil. Independent Study Project (ISP) Collection. http://digitalcollections.sit.edu/isp_collection/1944 (assessed on 10/10/2015).

[11] Conde MB, Melo FA, Marques AM, Cardoso NC, Pinheiro VG, Dalcin Pde T, et al (2009) III Brazilian Thoracic Association Guidelines on tuberculosis. J Bras Pneumol 35: 1018–1048. 19918635 10.1590/s1806-37132009001000011

[12] World Health Organization (2013) Systematic screening for active tuberculosis: principles and recommendations. Editor: World Health Organization. 25996015

[13] American Diabetes Society (2015) Diagnosing Diabetes and Learning About Prediabetes. http://www.diabetes.org/diabetes-basics/diagnosis/ (assessed on 07/10/2015).

[14] Alisjahbana B, Sahiratmadja E, Nelwan EJ, Purwa AM, Ahmad Y, Ottenhoff TH, et al (2007) The effect of type 2 diabetes mellitus on the presentation and treatment response of pulmonary tuberculosis. Clin Infect Dis 45: 428–435. 17638189 10.1086/519841

[15] Lohmann EM, Koster BF, le Cessie S, Kamst-van Agterveld MP, van Soolingen D, Arend SM (2012) Grading of a positive sputum smear and the risk of Mycobacterium tuberculosis transmission. Int J Tuberc Lung Dis 16: 1477–1484. 10.5588/ijtld.12.0129 22964038

[16] Mayer-Barber KD, Andrade BB, Oland SD, Amaral EP, Barber DL, Gonzales J, et al (2014) Host-directed therapy of tuberculosis based on interleukin-1 and type I interferon crosstalk. Nature 511: 99–103. 10.1038/nature13489 24990750 PMC4809146

[17] Pedrozo C, Sant'Anna C, de Fatima March M, Lucena S (2009) Clinical scoring system for paediatric tuberculosis in HIV-infected and non-infected children in Rio de Janeiro. Int J Tuberc Lung Dis 13: 413–415. 19275806

[18] Narayan S, Mahadevan S, Serane VT (2003) Keith Edwards score for diagnosis of tuberculosis. Indian J Pediatr 70: 467–469. 12921313 10.1007/BF02723134

[19] World Health Organization (2011) Collaborative Framework for care and control of tuberculosis and diabetes. http://www.who.int/diabetes/publications/tb_diabetes2011/en/ (assessed on 07/09/2015). 26290931

[20] Probst-Hensch NM (2010) Chronic age-related diseases share risk factors: do they share pathophysiological mechanisms and why does that matter? Swiss Med Wkly 140: w13072 10.4414/smw.2010.13072 20809438

[21] Uplekar MW, Rangan S, Weiss MG, Ogden J, Borgdorff MW, Hudelson P (2001) Attention to gender issues in tuberculosis control. Int J Tuberc Lung Dis 5: 220–224. 11326820

[22] Chiang CY, Bai KJ, Lin HH, Chien ST, Lee JJ, Enarson DA, et al (2015) The influence of diabetes, glycemic control, and diabetes-related comorbidities on pulmonary tuberculosis. PLoS One 10: e0121698 10.1371/journal.pone.0121698 25822974 PMC4378948

[23] Baker MA, Harries AD, Jeon CY, Hart JE, Kapur A, Lonnroth K, et al (2011) The impact of diabetes on tuberculosis treatment outcomes: a systematic review. BMC Med 9: 81 10.1186/1741-7015-9-81 21722362 PMC3155828

[24] Perez-Guzman C, Torres-Cruz A, Villarreal-Velarde H, Vargas MH (2000) Progressive age-related changes in pulmonary tuberculosis images and the effect of diabetes. Am J Respir Crit Care Med 162: 1738–1740. 11069805 10.1164/ajrccm.162.5.2001040

[25] Perez-Guzman C, Torres-Cruz A, Villarreal-Velarde H, Salazar-Lezama MA, Vargas MH (2001) Atypical radiological images of pulmonary tuberculosis in 192 diabetic patients: a comparative study. Int J Tuberc Lung Dis 5: 455–461. 11336277

[26] Bashar M, Alcabes P, Rom WN, Condos R (2001) Increased incidence of multidrug-resistant tuberculosis in diabetic patients on the Bellevue Chest Service, 1987 to 1997. Chest 120: 1514–1519. 11713128 10.1378/chest.120.5.1514

[27] Viswanathan V, Kumpatla S, Aravindalochanan V, Rajan R, Chinnasamy C, Srinivasan R, et al (2012) Prevalence of diabetes and pre-diabetes and associated risk factors among tuberculosis patients in India. PLoS One 7: e41367 10.1371/journal.pone.0041367 22848473 PMC3406054

[28] Gupta S, Shenoy VP, Bairy I, Srinivasa H, Mukhopadhyay C (2011) Diabetes mellitus and HIV as co-morbidities in tuberculosis patients of rural south India. J Infect Public Health 4: 140–144. 10.1016/j.jiph.2011.03.005 21843860

[29] Borekci S, Atahan E, Demir Yilmaz D, Mazican N, Duman B, Ozguler Y, et al (2015) Factors Affecting the Tuberculosis Risk in Patients Receiving Anti-Tumor Necrosis Factor-alpha Treatment. Respiration 90: 191–198. 10.1159/000434684 26137891

[30] Gray JM, Cohn DL (2013) Tuberculosis and HIV coinfection. Semin Respir Crit Care Med 34: 32–43. 10.1055/s-0032-1333469 23460004

[31] Syed Suleiman SA, Ishaq Aweis DM, Mohamed AJ, Razakmuttalif A, Moussa MA (2012) Role of diabetes in the prognosis and therapeutic outcome of tuberculosis. Int J Endocrinol 2012: 645362 10.1155/2012/645362 22570649 PMC3337603

[32] Chang JT, Dou HY, Yen CL, Wu YH, Huang RM, Lin HJ, et al (2011) Effect of type 2 diabetes mellitus on the clinical severity and treatment outcome in patients with pulmonary tuberculosis: a potential role in the emergence of multidrug-resistance. J Formos Med Assoc 110: 372–381. 10.1016/S0929-6646(11)60055-7 21741005

[33] Singla R, Khan N, Al-Sharif N, Ai-Sayegh MO, Shaikh MA, Osman MM (2006) Influence of diabetes on manifestations and treatment outcome of pulmonary TB patients. Int J Tuberc Lung Dis 10: 74–79. 16466041

[34] Reed GW, Choi H, Lee SY, Lee M, Kim Y, Park H, et al (2013) Impact of diabetes and smoking on mortality in tuberculosis. PLoS One 8: e58044 10.1371/journal.pone.0058044 23469139 PMC3585219

[35] Baghaei P, Marjani M, Javanmard P, Tabarsi P, Masjedi MR (2013) Diabetes mellitus and tuberculosis facts and controversies. J Diabetes Metab Disord 12: 58 10.1186/2251-6581-12-58 24360398 PMC7962555

